# Venous anomalies and thromboembolism

**DOI:** 10.1186/s12959-023-00484-5

**Published:** 2023-04-20

**Authors:** Caroline Dix, Warren Clements, Harry Gibbs, Joanne So, Huyen A Tran, James D McFadyen

**Affiliations:** 1 Department of Clinical Haematology, The Alfred Hospital, Melbourne, VIC 3004 Australia; 2 Department of Radiology, The Alfred Hospital, Melbourne, VIC 3004 Australia; 3 Department of General Medicine, Alfred Health, Melbourne, VIC 3004 Australia; 4 Australian Centre for Blood Diseases, Central Clinical School, Monash University, Melbourne, Australia; 5 Atherothrombosis and Vascular Biology Laboratory, Baker Heart and Diabetes Institute, Melbourne, 3004 Australia; 6 Baker Department of Cardiometabolic Health, University of Melbourne, Melbourne, Australia

**Keywords:** Venous thromboembolism, Anatomic variant, May-Thurner syndrome, Aneurysm, Anticoagulation, Endovascular, IVC agenesis

## Abstract

Patients with venous anomalies are at increased risk of developing venous thromboembolism (VTE) and subsequent complications, but they are often under-recognised. While unprovoked VTE may trigger testing for inherited thrombophilias and malignancy screening, anatomic variants are considered less often. Venous anomalies increase the risk due to venous flow disturbance, resulting in hypertension, reduced flow velocity and turbulence. Recognition is important as endovascular or surgical intervention may be appropriate, these patients have a high rate of VTE recurrence if anticoagulation is ceased, and the anomalies can predispose to extensive VTE and severe post-thrombotic syndrome (PTS). In this case series, we present representative cases and radiological images of May-Thurner syndrome (MTS), inferior vena cava (IVC) variants and venous aneurysms, and review the available literature regarding optimal diagnosis and management in each condition.

## Background

Venous thromboembolism (VTE) is a disorder with multiple causative factors, and which can result in significant morbidity. It has an annual incidence of 1–2 per 1000 persons per year, with a higher risk in those of increasing age and in males [1]. Virchow’s triad—venous stasis, hypercoagulability and endothelial dysfunction—has long been used to describe the pathophysiological underpinnings of VTE [2]. Risk factors for VTE reflect these pathophysiological processes, including immobility (venous stasis); trauma and surgery (endothelial dysfunction); and cancer, pregnancy and inherited thrombophilias (hypercoagulability) [3]. Unprovoked VTE often triggers testing for inherited thrombophilias, and appropriate malignancy screening, but anatomic variants of the venous circulation are considered less often. While uncommon, venous anomalies can result in an increased risk of VTE due to venous flow disturbance, which results in hypertension, reduced flow velocity, and turbulence. It is often under-recognised, particularly in young patients with unprovoked VTE, or VTE in unusual locations [4, 5]. Recognition is important for three major reasons – firstly, there may be surgical or endovascular options available to reconstruct and/or address a potentially reversible anomaly; secondly the risk of recurrence is high, and these patients may require indefinite anticoagulation; and thirdly, some of these anomalies predispose to extensive VTE and may result in phlegmasia cerulea dolens or severe post-thrombotic syndrome (PTS) [6]. Here we present a case series of different anatomic variants – May-Thurner syndrome, inferior vena cava (IVC) abnormalities, and venous aneurysms—resulting in VTE, and discuss the available literature with regards to optimal diagnostic strategies and management.

## May-Thurner syndrome

May-Thurner syndrome (MTS) is characterised by extrinsic compression of a vein by the arterial system against bony structures in the iliocaval territory resulting in venous outflow obstruction; the original description and the most common abnormality involves the left iliac vein being compressed by the right iliac artery against a vertebrae [7, 8]. Its exact incidence is unknown but thought to affect 2–5% of those with a deep vein thrombosis (DVT) [9]. We report on two cases of MTS.

**Fig. 1:**
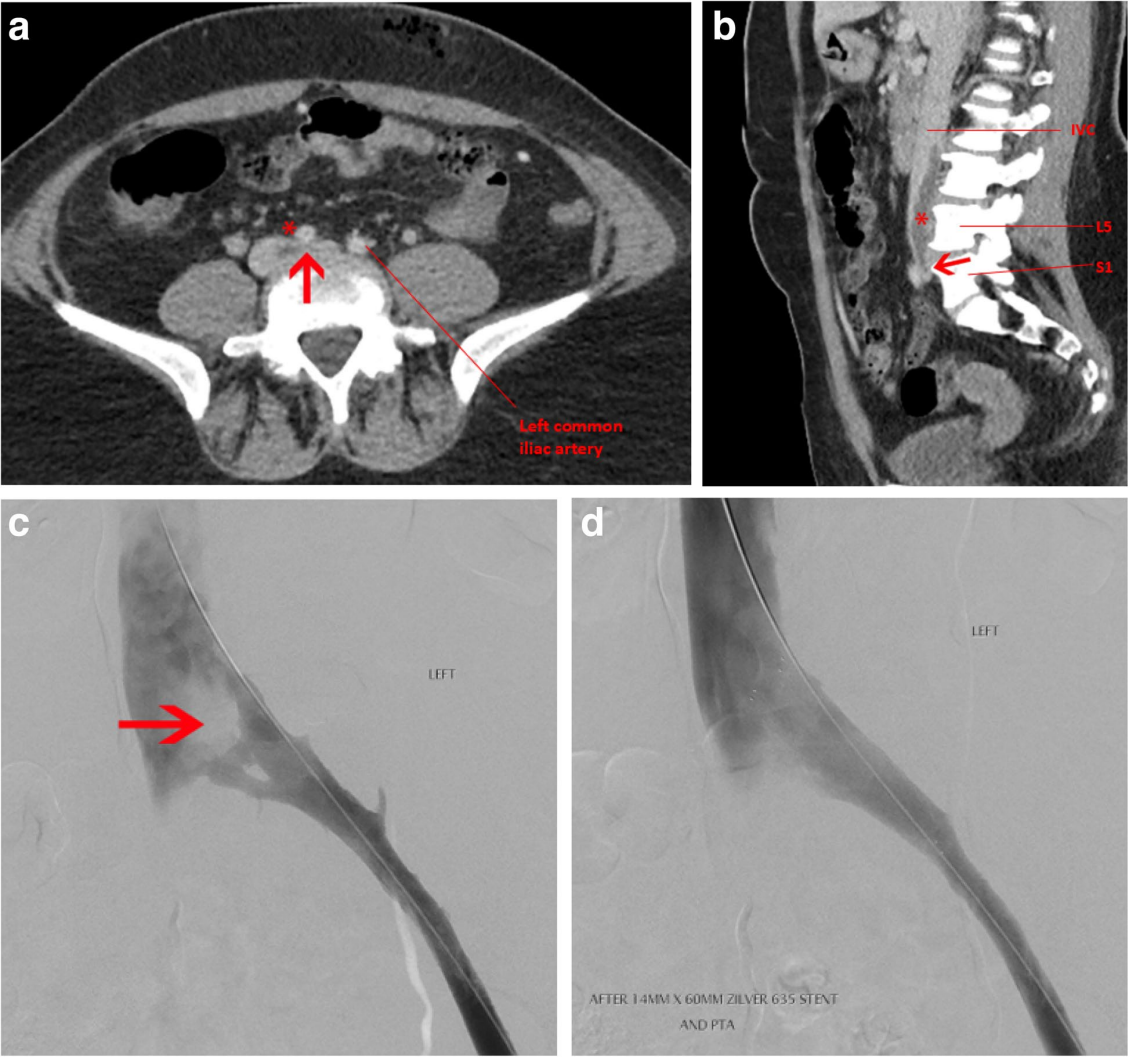
**a** Anatomy seen in May-Thurner Syndrome: Axial CT venogram of the pelvis shows compression of the left common iliac vein (arrow) between the right common iliac artery (asterisk) and the intervertebral disc of L5-S1. **b** Anatomy seen in May Thurner Syndrome: Sagittal CT venogram of the pelvis shows compression of the left common iliac vein (arrow) between the right common iliac artery and the intervertebral disc of L5-S1, with acute thrombus in the lower IVC (asterisk). **c** Frontal iliocaval angiography shows extrinsic compression of the left common iliac vein (arrow). This anatomy is seen in patients with May-Thurner Syndrome. **d** After placement of self-expanding 14 mm venous stent, improvement in flow is seen as well as resolution of extrinsic compression on the left common iliac vein

The first is a 17-year-old female who presented initially with an unprovoked left proximal DVT treated with warfarin for 6 months. She had a symptomatic recurrence 6 months after anticoagulant cessation, with ultrasound and CT venogram confirming left common iliac vein compression by the right common iliac artery (Fig. 1a). She was anticoagulated with apixaban with a plan to continue this long-term. She had moderate PTS, managed with a compression stocking. The second is an 18-year-old female who presented with a painful, oedematous left leg on a background of being on the combined oral contraceptive pill (COCP) for 2 years. She was found to have occlusive thrombus in the femoral vein, extending proximally into the external iliac vein and lower IVC (Fig. 1b). She underwent catheter-directed thrombolysis and was found to have a left common iliac vein stenosis, the combination of findings diagnostic of MTS. A stent was placed across the stenosis (Fig. 1c and d) and she remains on long-term oral anticoagulation without VTE recurrence and no evidence of PTS.

MTS results in focal, intermittent compression of the vein, leading to endothelial injury and impaired venous return from the ipsilateral limb [4]. The exact incidence is unknown, and establishing this is further hampered by the lack of a precise definition for the degree of left common iliac vein compression required [4]. A recent Australian study identified the required degree of compression that can be used to correlate to the diagnosis of MTS, however the authors noted that the same degree of compression can be seen in the general population and that compression alone does not constitute a diagnosis of MTS [10].

Estimates of incidence range from 2–5% of all-comers with DVTs, to over 30% of those with left-sided or bilateral lower limb thromboses [9]. Fibrous spurs consistent with MTS have been found in 22–33% of cadavers [8]. Therefore, many individuals with MTS anatomy do not suffer from venous thrombosis, and it likely depends on both the degree of compression and the presence of additional risk factors [4]. MTS is more common in women than men, the explanation for which is not entirely clear but is potentially related to accentuation of the lumbar lordosis in the female pelvis which pushes the lower lumbar vertebrae anteriorly thus accentuating compression of the left common iliac vein [11].

The role of endovascular treatment in MTS is contentious. Options include catheter-directed thrombolysis, balloon angioplasty and/or stent placement; these are associated with low morbidity, a high stent patency rate and may allow patients to cease anticoagulation following stent endothelialisation [4]. Endovascular treatment traditionally has been thought to reduce the rate of PTS and improved quality of life, but a more recent randomised controlled trial involving patients with proximal DVT (i.e. not specific for those with MTS) cast doubt over this, finding that thrombolysis had no benefit over anticoagulation alone in regards to rate or severity of PTS [12–14]. Iliac stenting may be considered in those with established PTS in the setting of MTS, with studies showing improvement in PTS symptoms and hastened healing of venous ulcers in advanced PTS [15–17]. There have been no randomised trials regarding outcomes of stenting iliac vein lesions compared to conservative management (anticoagulation alone) in MTS, and the decision to place a stent should take into account many factors including clinical context, likelihood the lesion explains the symptoms and signs, severity of venous disease and patient preference [18].

## Inferior Vena Cava (IVC) variants

**Fig. 2:**
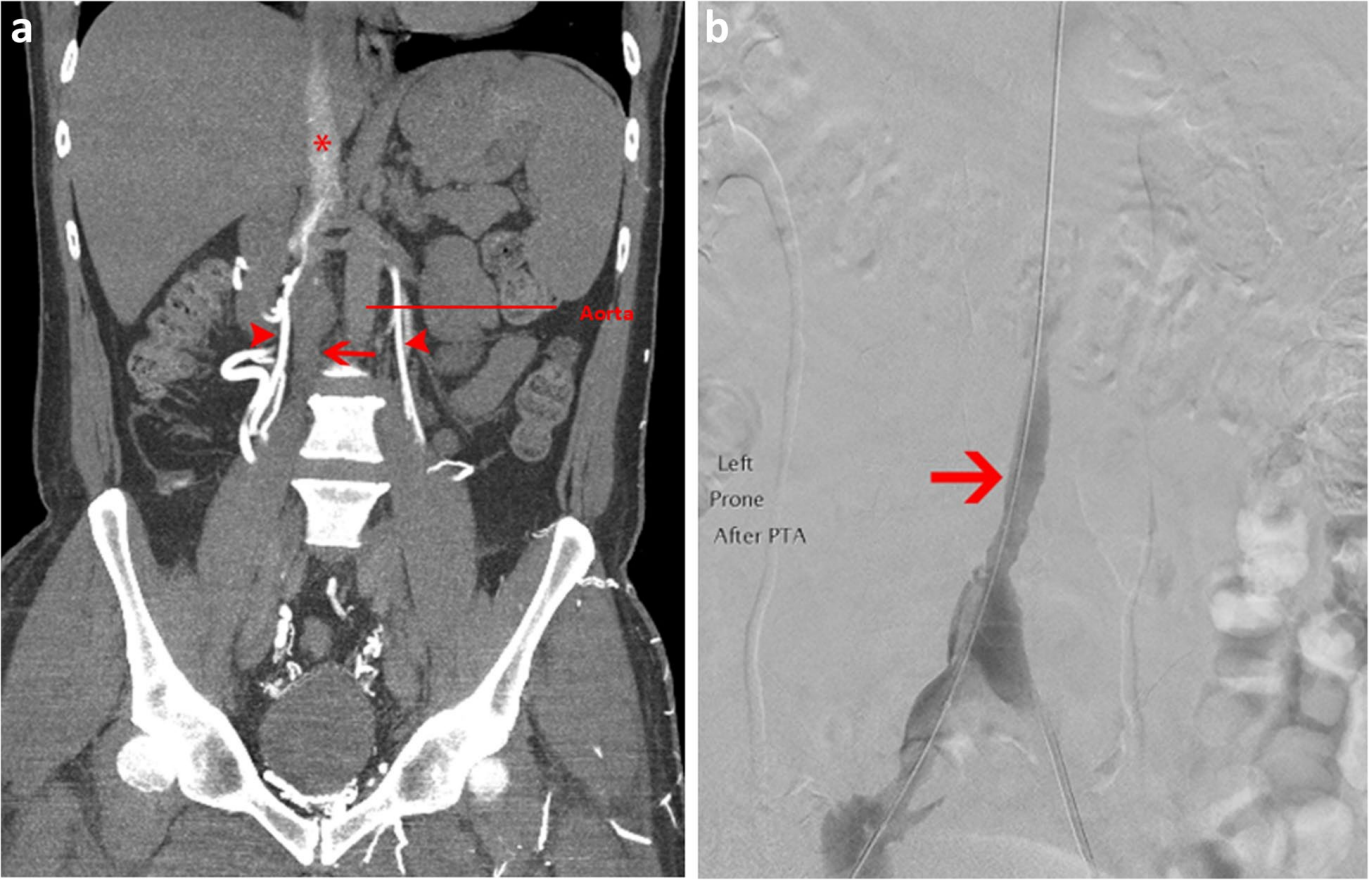
**a** Coronal CT venogram shows hypoplastic and acutely thrombosed infrarenal IVC (arrow) with contrast taking path of mature iliolumbar and lumbar collateral veins (arrow head). The suprarenal IVC is patent (asterisk). **b** Venogram after thrombolysis shows restoration of flow to the infrarenal IVC which remains hypoplastic (arrow)

Congenital anomalies of the IVC are rare abnormalities that can predispose to lower limb DVT due to venous hypertension and chronic reduction in central venous velocity. We present two cases of DVTs in individuals with IVC variants. The first is a 36-year-old female presenting with right leg pain and oedema while on the COCP, with an ultrasound showing extensive thrombus extending from the calf into the IVC. A CT venogram found a hypoplastic and thrombosed infrarenal IVC (Fig. 2a). She had large collaterals filling the azygos system, consistent with a congenital anomaly of the IVC. She underwent thrombolysis and suprarenal balloon angioplasty of the stenosed segment but was unsuitable for a stent (Fig. 2b). She remains on long term oral anticoagulation with mild PTS.

**Fig. 3:**
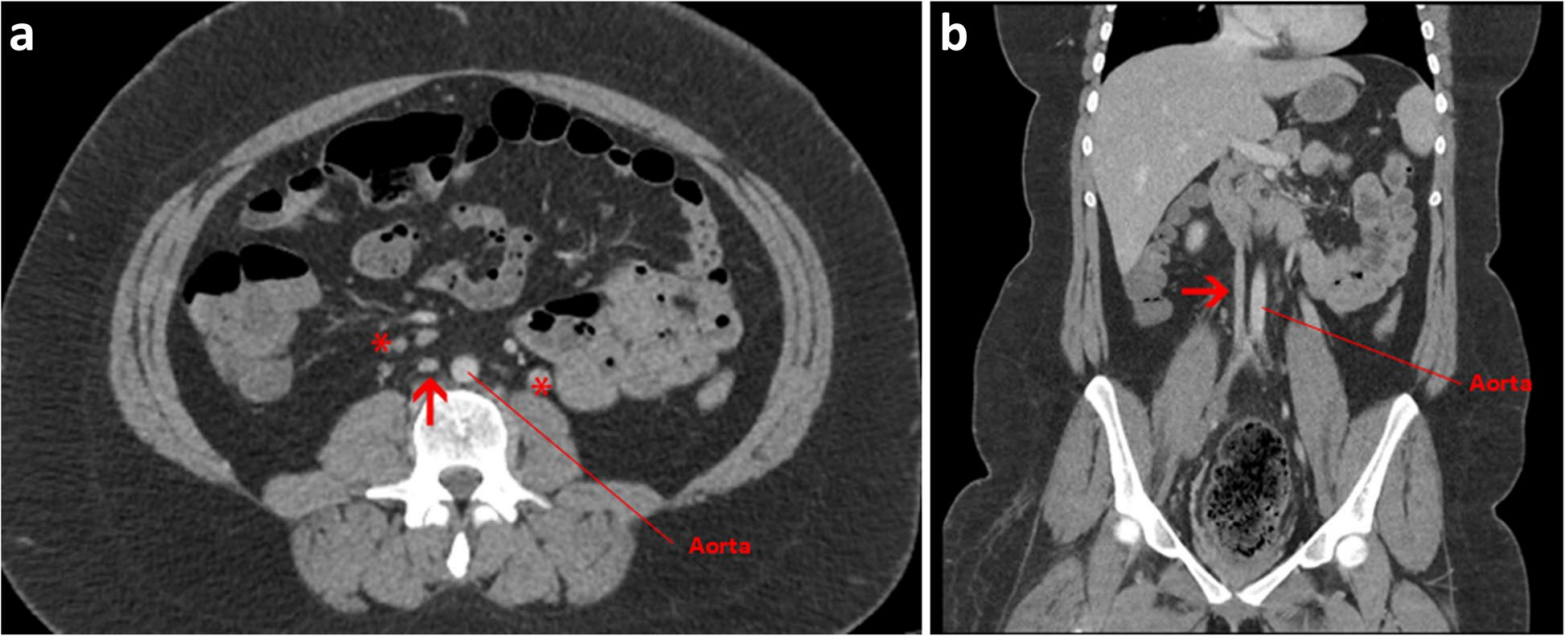
**a** Axial CT venogram shows small calibre IVC over a long distance (arrow) and established collateral veins (asterisks) compatible with hypoplastic IVC. **b** Coronal CT venogram shows small calibre IVC over a long distance (arrow) compatible with hypoplastic IVC

The second is a 60yo male found to have an extensive proximal right leg DVT, extending from the iliac vein proximally to the popliteal vein distally. This occurred on a background of having had a left leg DVT ~ 20 years prior, treated with warfarin for 10 years. On re-review of a CT from 20 years prior, he was found to have a hypoplastic IVC with multiple retroperitoneal collateral vessels (Fig. 3a-b). He was strongly advised to take long-term oral anticoagulation, but he self-ceased ~ 12 months after the DVT. He then re-presented with right leg discomfort and was found to have extensive DVT into the iliac vein. He now remains on lifelong oral anticoagulation and has moderate PTS.

IVC agenesis is a rare anomaly, with a large registry study of over 50,000 patients with lower limb DVT finding IVC agenesis in 0.06%, although higher rates of up to 8.7% in the general population have been reported [5, 19–21]. IVC agenesis is likely caused by either aberrant development during embryogenesis or potentially intrauterine or perinatal thrombosis of the IVC with subsequent obliteration of the vein [19]. A collateral venous circulation develops as an adaptive response, which may explain why these findings are often incidental. Due to turbulent flow, they remain a persistent risk factor for the development of VTE in the lower limbs [22]. Co-existent pulmonary embolism (PE) is rare, since thrombi are trapped within collateral circulations (such as azygos vein) before reaching the pulmonary circulation [21, 23]. Young age and unprovoked DVT, especially if proximal and bilateral, should raise suspicion of IVC agenesis [21]. In contrast to MTS which predominantly affects women, IVC agenesis and hypoplasia is more common in males [22, 24]. Interestingly, despite the fact that individuals have had the variant lifelong, they often do not present until the third or fourth decade, there is probably an additional “trigger” for the thrombosis, such as starting the COCP in a female; one report suggests DVT may follow intensive and unusual physical activity as the collaterals can increase blood flow which then generates venous stasis within the IVC segment [23, 25]. The gold standard modality for assessment is either CT or MRI. Optimal management has yet to be defined, with lifelong anticoagulation, thrombolysis and/or surgical thrombectomy all potentially useful depending on the individual circumstances. Studies have shown a low risk of recurrence if anticoagulation is continued, and the finding of an IVC abnormality is a strong indication for extended anticoagulation [22]. Patients with IVC variants have a high risk of PTS, reported to affect up to 50% of patients, probably related to the size and extent of the DVT [21, 22]. Further studies are required into the optimal management of these patients.

## Venous aneurysms

**Fig. 4:**
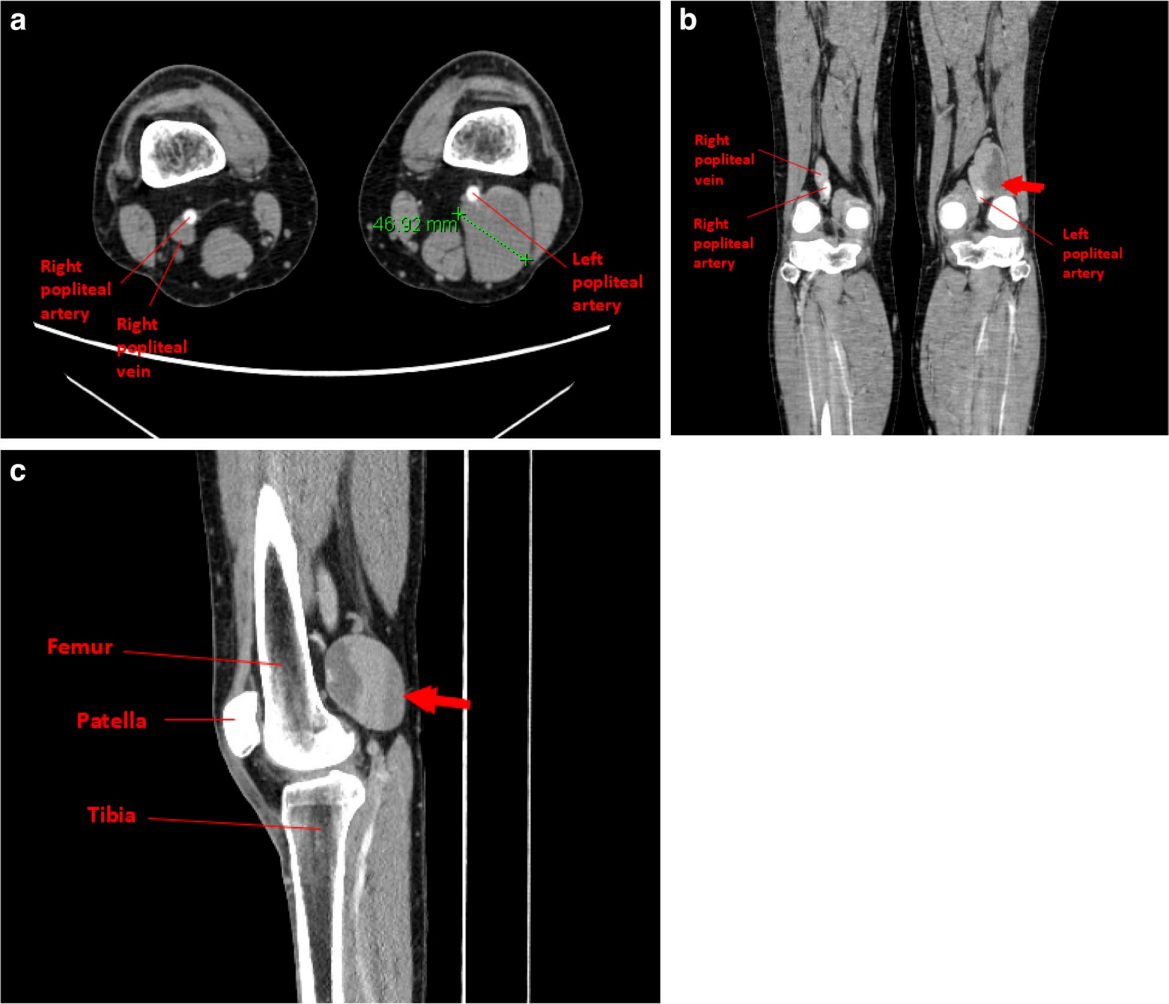
**a** axial CT angiogram showing left popliteal vein aneurysm measuring 46.92 mm. **b** coronal image showing left popliteal vein aneurysm (arrow), with mural thrombus within aneurysmal segment. **c** Sagittal CT image showing left popliteal vein aneurysm (arrow)

Venous aneurysms are another abnormality which can predispose to thrombosis due to flow disturbance. We present a case of a 57yo male with an unprovoked sub-massive PE, which was managed with warfarin. Two years later, he presented with new acute PEs despite consistently therapeutic INRs, resulting in a cardiac arrest and requiring thrombolysis. His anticoagulation was changed to low molecular weight heparin. He was then found to have a left popliteal vein aneurysm with mural thrombus within the aneurysmal segment, on routine screening lower limb doppler ultrasound and confirmed on CT (Fig. 4a-c). He had an IVC filter inserted and the aneurysm was subsequently repaired. He continued oral anticoagulation long-term without any further thrombotic episodes.

In a case of a venous aneurysm in a different location, a 59yo female who was found to have extensive collateral and intra-abdominal varices on a CT scan of her upper abdomen performed for chronic abdominal pain. There was no evidence of structural liver disease. A doppler ultrasound subsequently identified a portal vein aneurysm, confirmed on catheter angiography. Twelve months later, on routine surveillance ultrasound, she was found to have a portal vein thrombus with extension in the superior mesenteric and splenic veins. Extensive thrombophilia testing, including for JAK2, antiphospholipid syndrome and paroxysmal nocturnal haemoglobinuria, was negative. She was treated with a DOAC and has been advised to continue this long-term.

Venous aneurysms are uncommon vascular malformations, defined as a solitary dilatation of a venous segment that communicates with a main venous structure by a single channel [26]. They can be considered primary, or secondary to events such as trauma, surgery or infection (which would be classified as pseudoaneurysms) [27]. Aneurysms of the deep venous system can increase the risk of VTE due to altered flow within the aneurysmal segment. Deep venous aneurysms may be identified after investigations for VTE, and can be diagnosed by doppler ultrasonography, CT venography or MRI. There is a paucity of literature on the optimal management, but most reports suggest surgical management is appropriate in the majority of cases, as well as long-term anticoagulation if there are no contraindications [26].

Portal vein aneurysms require special mention as they are often found incidentally on investigation of abdominal pain but can increase the risk of portal vein thrombosis. They are rare and can be congenital or acquired, with portal hypertension/cirrhosis the most common cause of acquired portal vein aneurysms. Other causes include severe pancreatitis, trauma and malignancy [28]. 50% present with non-specific abdominal pain, around one-third are discovered incidentally, and the remainder presenting with symptoms due to compression of adjacent structures or gastrointestinal bleeding [28]. Portal vein thrombosis is reported to affect around 20% of those with a portal vein aneurysm [28]. Currently surgical management is only considered if there are complications such as rupture or at risk of rupture due to size, or pain related to the aneurysm [28].

## Conclusion

Venous anatomical variants, including MTS, IVC abnormalities and venous aneurysms, are an under-recognised contributor to venous thromboembolic disease. They should be considered in young patients who present with unprovoked VTE, particularly extensive proximal DVTs or if there are unusual features (such as bilateral DVT in IVC agenesis, or breakthrough VTE while on therapeutic anticoagulation). Identification is important as there is a high risk of recurrence if anticoagulation is ceased, and because surgical management may be an option.

## Data Availability

The information used for the current study may be available from the corresponding authors on reasonable request.
